# Antigenic escape is accelerated by the presence of immunocompromised hosts

**DOI:** 10.1098/rspb.2022.1437

**Published:** 2022-11-09

**Authors:** Ryuichi Kumata, Akira Sasaki

**Affiliations:** Department of Evolutionary Studies of Biosystems, The Graduate University of Advanced Studies, SOKENDAI, Hayama, Kanagawa 2400139, Japan

**Keywords:** antigenic escape, host heterogeneity, immunodeficiency, travelling wave

## Abstract

The repeated emergence of SARS-CoV-2 escape mutants from host immunity has obstructed the containment of the current pandemic and poses a serious threat to humanity. Prolonged infection in immunocompromised patients has received increasing attention as a driver of immune escape, and accumulating evidence suggests that viral genomic diversity and emergence of immune-escape mutants are promoted in immunocompromised patients. However, because immunocompromised patients comprise a small proportion of the host population, whether they have a significant impact on antigenic evolution at the population level is unknown. We consider an evolutionary epidemiological model that combines antigenic evolution and epidemiological dynamics. Applying this model to a heterogeneous host population, we study the impact of immunocompromised hosts on the evolutionary dynamics of pathogen antigenic escape from host immunity. We derived analytical formulae of the speed of antigenic evolution in heterogeneous host populations and found that even a small number of immunocompromised hosts in the population significantly accelerates antigenic evolution. Our results demonstrate that immunocompromised hosts play a key role in viral adaptation at the population level and emphasize the importance of critical care and surveillance of immunocompromised hosts.

## 1. Introduction

The repeated emergence of new variants hinders the control of epidemics [1–3]. By acquiring mutations to escape existing adaptive immunity, which works in the strain-specific manner, pathogens can continue to thrive in host populations. The current pandemic of SARS-CoV-2 is due to the repeated emergence and spread of variants with undesirable phenotypes, including increased infectivity and high mortality, known as VOCs (variants of concern) [3–5]. Each time an existing strain is replaced by a VOC, the number of cases and deaths increases leaving a significant impact on society in terms of both public health and the economy. Accumulating evidence indicates that each VOC evades host immunity developed by previous infections with pre-existing strains and vaccines [6,7] and that multiple mutations in the S-gene region targeted by host immunity contribute to immune escape [8]. Antigenic escapes from the host immune system by changing their antigenicity have also been observed in other viruses including HIV-1 and influenza virus [9–12]. Thus, the emergence of new variants is undoubtedly an important factor in determining the dynamics of infectious diseases.

Prolonged and persistent infection may facilitate the emergence of variants of SARS-CoV-2 [13,14]. Because mutations occur as pathogens replicate in the host body, the emergence of a new variant depends on the within-host dynamics. Pathogens that successfully infect the host are usually quickly eliminated by the immune system of the host. However, in hosts with compromised immune function due to medical treatment or diseases such as AIDS, the elimination of pathogens may be delayed, resulting in prolonged infection. Recently, long-term infection with SARS-CoV-2 and influenza virus in immunocompromised patients has been reported [15–23]. The longer a pathogen stays in an infected person, the more it replicates, increasing the probability of mutation. Thus, persistent infection likely influences the emergence of new mutations (other possible mechanisms to promote viral mutation are discussed later). Indeed, in the case of SARS-CoV-2 and influenza A virus, the accumulation of mutations and high genomic diversity within immunocompromised hosts has been reported [20–23], suggesting that an immunocompromised host may increase the likelihood of mutation. Therefore, host heterogeneity in the time from infection to recovery may affect the accumulation of undesirable mutations.

Although evidence for the promotion of mutations in immunocompromised hosts is accumulating, the extent to which the presence of immunocompromised hosts affects antigenic escape on a population scale remains unclear. Moreover, immunocompromised patients are rare, and whether the small number of such patients has a significant impact on viral immune escape at the population level has yet to be elucidated. In this study, we theoretically examine the evolutionary dynamics of pathogen antigenic escape in a host population with heterogeneity in recovery rates from infection. We then aim to determine how such heterogeneity affects epi-evolutionary dynamics at the population level.

Several theoretical studies have attempted to elucidate the complex evolutionary epidemiological dynamics in which pathogens continue to evade host immunity by repeatedly changing their antigenicity [24–29]. These studies are motivated by rapid antigenic drift (repeated escapes of viral antigenicity from host immunity) of influenza A viruses at host population level [30], the antigenic drift of HIV and HCV [31,32] within patients with AIDS and hepatitis C, and antigenic switching of trypanosomes in patients with sleeping disease [33]. Focusing on the antagonistic interaction between host immunity and pathogen variants, the evolutionary escape of pathogen antigenicity and the specific adaptive immunity in the host are mathematically analysed as the joint ‘travelling waves’ of pathogen antigenicity and host immunity along antigenicity space where pathogen ‘movements’ are due to random mutations [24–26,28], rather than the dispersal in physical space on which most biological travelling waves were analysed [34–36]. These studies have revealed the speed of antigenic escape [26–28] and predicted cross-immunity-driven discontinuous outbreaks in both time and antigenic space [24–26]. They also evaluated the joint evolution of pathogen virulence and antigenic escape [25]. However, few studies have examined the epidemiological dynamics of antigenic escape when heterogeneity in host traits, such as recovery rates, is present. Smith & Ashby's [37] stochastic simulations suggest that the presence of hosts with low recovery rates increases the likelihood of overcoming the fitness landscape valley, but the impact of heterogeneity in host recovery rates on antigenic escape rates and epidemics in more general situations remains unresolved.

This study aims to examine the impact of host heterogeneity in the recovery rate on the population-level dynamics of antigenic escape of pathogens. We construct a simple mathematical model that accounts for host heterogeneity and investigate the effect of heterogeneity on the evolutionary epidemiological dynamics of pathogen antigenicity. We then study how the rate of antigenic escape, i.e. the rate of the accumulation of antigen-changing mutations in a pathogen, is affected by host heterogeneity. We also examine how epidemic control measures affect the speed of antigenic escape and discuss implications for efficient control measures to slow the accumulation of immune-escape mutants.

## 2. Model

**Figure 1.**
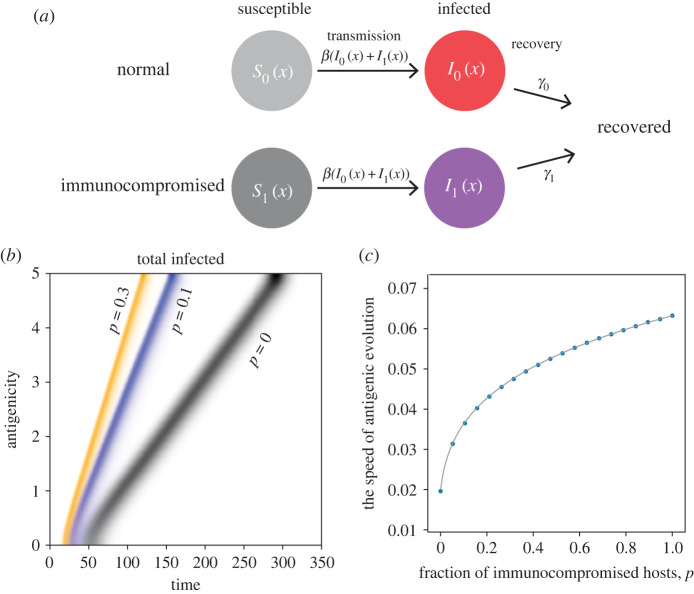
Antigenic drift in the population with heterogeneity in immune competency. (*a*) A graphical representation of the epidemiological model in the presence of immunocompromised hosts. (*b*) Changes in the distribution of antigenicity over time obtained by numerical simulations of the model (1) with different proportions of immunocompromised hosts (shown by different colours). Antigenic drift in the population consisting only of hosts with normal immune competency (*p* = 0) is shown in black, while that of populations where 10% and 30% of hosts are immunocompromised is shown in blue and orange, respectively. (*c*) The speed of antigenic evolution as a function of the fraction *p* of immunocompromised hosts. The dots show the speeds obtained by numerical simulations of the model (1), and the solid curve is the analytical prediction calculated from equation (2.3). Parameters are *β* = 1.1, *γ*_0_ = 1, *γ*_1_ = 0.1 and *D* = 0.001. (Online version in colour.)

We consider a host population made up of two classes of individuals differing in their recovery rates from an infectious disease (figure 1*a*). Specifically, if we denote the immunocompromised hosts as class 1 and the remaining individuals as class 0, the recovery rate of class 1 is significantly smaller than that of class 0 (or the mean infectious period is significantly longer in class 1 than in class 0). We consider the epidemiology and evolution of antigenic variants in such a heterogeneous host population under the assumption that antigenic phenotypes can be indexed in a one-dimensional space.

We denote *S_i_*(*t*, *x*) as the proportion of class *i* hosts that are susceptible to the antigenic variant *x* of the pathogen at time *t*. *I_i_*(*t*, *x*) represents the proportion of class *i* hosts that are infected by the antigenic variant *x* at time *t*. The class *i* hosts that are susceptible to the antigenic variant *x* become infected with the force of infection *β*(*I*_0_(*t*, *x*) + *I*_1_(*t*, *x*)) per unit time, where *β* is the transmission rate of the pathogen. We assume that the pathogen variants have identical traits except for antigenicity. An epidemic from an antigenic variant of a pathogen will end as the proportion of hosts susceptible to the variant is decreased. However, an immune-escaping variant can be generated through mutation before the focal epidemic is completely faded out to start another epidemic from the new variant. Random generation of antigenic mutants can be introduced by the diffusion term below, with diffusion constant *D* in the dynamics [24–26,28]. The infected hosts in class *i* then recover with the recovery rate *γ_i_*. Combining these epi-evolutionary processes, the proportions *S*_0_(*t*, *x*), *S*_1_(*t*, *x*), *I*_0_(*t*, *x*) and *I*_1_(*t*, *x*) change with time as follows:

2.1 $$\begin{array}{rl} \frac{\partial S_{0}(t,x)}{\partial t}= & -\beta S_{0}(t,x)(I_{0}(t,x)+I_{1}(t,x)),\\ \frac{\partial S_{1}(t,x)}{\partial t}= & -\beta S_{1}(t,x)(I_{0}(t,x)+I_{1}(t,x)),\\ \frac{\partial I_{0}(t,x)}{\partial t}= & \beta S_{0}(t,x)(I_{0}(t,x)+I_{1}(t,x))-\gamma_{0}I_{0}(t,x)+D\frac{\partial^{2}I_{0}(t,x)}{\partial x^{2}}\\ \mathrm{and}\;\frac{\partial I_{1}(t,x)}{\partial t}= & \beta S_{1}(t,x)(I_{0}(t,x)+I_{1}(t,x))-\gamma_{1}I_{1}(t,x)+D\frac{\partial^{2}I_{1}(t,x)}{\partial x^{2}}. \end{array}$$

Though this is an SIR model (a compartment model for the changes in susceptible, infected and recovered hosts densities), the proportions of the recovered or dead individuals are omitted as they do not affect the dynamics for *S_i_* or *I_i_*. The diffusion coefficient *D* is calculated as the product of the mutation rate *μ* and the mean-squared deviation of mutational change Δ*x* in antigenicity, *η*^2^ = *E*[(Δ*x*)^2^], using the equation *D* = *μη*^2^/2. The loss by recovery term in this model can be interpreted as any event responsible for the termination of infectiousness, including death caused by infection. In this paper, we assume that an immunocompromised host has an *m* times longer mean infectious period (time to recovery) than other hosts in the following equation:

$$\frac{1}{\gamma_{1}}=m\frac{1}{\gamma_{0}},$$

where *m* >1. Another important parameter of the model is the fraction *p* of immunocompromised hosts in the population before the epidemic. Therefore, the initial proportion of hosts in each class at time *t* = 0 that are susceptible to a pathogen variant of antigenicity *x* is *S*_0_(0, *x*) = 1 − *p* and *S*_1_(0, *x*) = *p*, respectively. The present model (1) does not take into account the cross-immunity between different antigenic variants. The extended analysis of the model including cross-immunity is discussed in electronic supplementary material, Information.

### (a) Antigenic escape in a homogeneous host population

Before showing the results of model (1), we briefly summarize how antigenic drift proceeds in a homogeneous host population where there is no host class 1 (*p* = 0) and all individuals have the same recovery rate *γ*_0_. The dynamics (1) are then reduced to

$$\begin{array}{rl} \frac{\partial S_{0}(t,x)}{\partial t}= & -\beta S_{0}(t,x)I_{0}(t,x)\\ \mathrm{and}\;\frac{\partial I_{0}(t,x)}{\partial t}= & \beta S_{0}(t,x)I_{0}(t,x)-\gamma_{0}I_{0}(t,x)+D\frac{\partial^{2}I_{0}(t,x)}{\partial x^{2}}. \end{array}\}$$

When a small amount of the pathogen variant with antigenicity *x* = 0 is introduced into the host population fully susceptible to the pathogen (*S*_0_(0, *x*) = 1 for all *x* and *I*_0_(0, *x*) = *ɛδ*(*x*), where *ɛ* is a small positive constant and *δ*(*x*) is the Dirac delta function), the pathogen persists by continuously evading host immunity as a travelling wave in antigenic space [24–26,28,34,38,39] with a constant wave speed:

2.2 $$\begin{array}{c} v_{0}=2\sqrt{(\beta -\gamma_{0})D}. \end{array}$$

In other words, the speed of accumulation of antigenic mutants asymptotically approaches two times the geometric mean of the pathogen's initial growth rate *β* − *γ*_0_ in a disease-free population and the diffusion constant *D* (half of the mutation variance). The total proportion of infected hosts maintained in the travelling wave $\Phi =\int I_{0}(t,x)\;dx$ approaches a constant determined from the following:

2.3 $$\begin{array}{c} \Phi =\frac{v_{0}}{\gamma_{0}}\left(1-\exp\left[-\frac{\beta }{v_{0}}\Phi \right]\right) \end{array},$$

[28]. Our aim in this paper is to extend these results to cases where the host population is heterogeneous to examine the effect on the speed of antigenic escape.

## 3. Results

### (a) Antigenic escape in a heterogeneous host population

The epi-evolutionary dynamics of antigenic escape of pathogens in a heterogeneous host population is studied using model (1). When a small number of hosts infected by a pathogen variant with a certain antigenicity (*x* = 0) is introduced into a disease-free host population, the primary outbreak occurs with the antigenic variant *x* = 0 providing that its basic reproduction number is greater than 1. Before the pathogen is cleared from the population from increased herd immunity, an antigenic mutant that can sufficiently escape host herd immunity raised by the primary outbreak could start the next outbreak. The sequence of such antigenic escapes by the pathogen and chase by host immunity could lead to the persistent epidemic of pathogens continuously changing their antigenicity (antigenic drift). The antigenic drift of pathogens can be modelled mathematically as coupled travelling waves of antigenic variants of the pathogen and antigen-specific immunity of a homogeneous host population [24–26,28]. Our model with host heterogeneity in immune competency (the ability of immune system to respond to antigenic stimulation) showed stable coupled travelling waves of pathogen antigenic variants and specific host immunity in the antigenic space with a constant speed (figure 1*b*) as in the previous models [24–26,28]. However, we found that the wave speed, which corresponds to the speed of the accumulation of antigenic mutations, i.e. the pathogen's speed of notorious evolutionary changes against host immunity and vaccination, depends on the host heterogeneity in immune competency (figure 1*c*), which is detailed in the rest of the paper.

The width of the cross-immunity indicates the extent to which specific immunity raised at one antigenicity is effective against a similar antigenicity. This measure of immune specificity affects the dynamical behaviour of pathogen's antigenic escape. If the cross-immunity width is sufficiently narrow, pathogen continuously escapes its antigenicity, and the host immunity follows it closely (exemplified as a smooth travelling wave with a constant wave speed as shown in figure 1 where cross-immunity width is assumed to be infinitesimally narrow, i.e. immunity is strictly specific). However, when the width of cross-immunity exceeds a certain threshold, the smooth travelling wave becomes unstable and sudden pathogen outbreaks are periodically repeated with long silent periods, accompanied by discontinuous antigenicity shift [24–26]. Such large shifts in antigenicity are also observed in our model with host heterogeneity in immune competency, and the periods in time and antigenic space between adjacent outbreaks depend on the heterogeneity (electronic supplementary material, figure S1). While such discontinuous antigenic escape is phenomenologically and practically important, the speed of the coupled travelling waves, which is our focus in this paper, is determined by the linearized system at the front edge of the travelling wave. Thus, the speed of travelling wave does not change by adding the cross-immunity structure.

### (b) Speed of antigenic escape and sensitivity to host heterogeneity

The linear analysis of the system (1) at the frontal end of the travelling wave of antigenic variants found in the Methods section reveals the analytical formulae for the wave speed *v* in the heterogeneous host population. The wave speed *v* in the heterogeneous host population consisting of the fraction 1 − *p* of hosts with a normal recovery rate *γ*_0_ and the fraction *p* of hosts with a lower recovery rate *γ*_1_ is given explicitly as follows:

3.1 $$\begin{array}{c} v=\sqrt{2D\left(\beta -(\gamma_{0}+\gamma_{1})+\sqrt{{\left(\beta -\gamma_{0}+\gamma_{1}\right)}^{2}+4\beta (\gamma_{0}-\gamma_{1})p}\right)}. \end{array}$$

We assume that *β* > *γ*_0_ > *γ*_1_. This reduces to $v_{0}=2\sqrt{(\beta -\gamma_{0})D}$ if *p* = 0, and to $v_{1}=2\sqrt{(\beta -\gamma_{1})D}$ if *p* = 1, which match the expressions previously demonstrated to model the travelling waves in the homogeneous host population. The analytical formula (3.1) perfectly matches the wave speeds observed in the numerical simulation of (1) for varied *p* (figure 1*c*).

**Figure 2.**
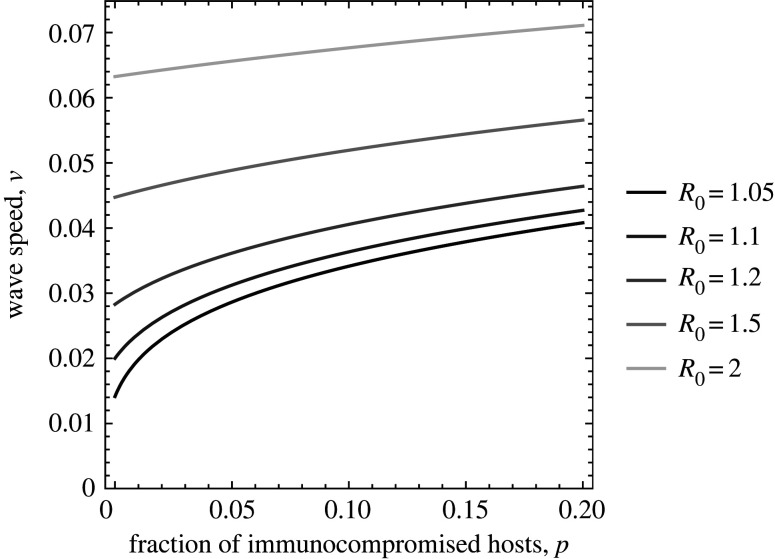
The sensitivity of wave speed *v* to the fraction of immunocompromised hosts. The curve is the wave speed calculated from (2.3). Different colours represent different *R*_0_ = *β*/*γ*_0_ values. The other parameters are *γ*_0_ = 1, *γ*_1_ = 0.1 and *D* = 0.001. (Online version in colour.)

Equation (3.1) reveals several important results. First, the wave speed *v* of antigenic escape monotonically increases with the fraction *p* of immunocompromised hosts from *v* = *v*_0_ at *p* = 0 to *v* = *v*_1_ at *p* = 1. Second, the wave speed (3.1) is a saturating (or concave) function of *p*. Thus, the smaller the fraction of immunocompromised hosts, the larger its accelerating effect on the wave speed (figure 2). This has important implications for epidemic forecasting and its control, given the small proportion of immunocompromised hosts in most communities. Third, the closer the basic reproduction number is to 1 (*R*_0_ = *β*/*γ*_0_ of the pathogen in the population consisting only of hosts with normal immune competency), the more sensitively the wave speed *v* increases with *p* (figure 2). This is most drastically shown with maximum sensitivity, (d*v*/d*p*)*_p_*_=0_, of the speed of antigenic escape to the proportion *p* of immunocompromised hosts at *p* = 0 (figure 2), calculated from (3.1):

3.2 $$\begin{array}{c} {\alpha =\frac{dv}{dp}|}_{\;p=0}=\frac{(m-1)R_{0}\sqrt{\gamma_{0}D}}{[m(R_{0}-1)+1]\sqrt{R_{0}-1}}, \end{array}$$

which diverges to infinity as the basic reproduction number *R*_0_ = *β*/*γ*_0_ approaches 1, where *m* = *γ*_0_/*γ*_1_ > 1 is the ratio of the infectious periods of immunocompromised hosts to that of hosts with normal immune competency. To summarize, the speed of the accumulation of antigenic escape mutants is sensitive to the presence of immunocompromised hosts: the speed increases sensitively with the frequency of immunocompromised hosts when they are rare (figure 1*c* and 2).

### (c) Impact of immunocompromised hosts on disease dynamics

**Figure 3.**
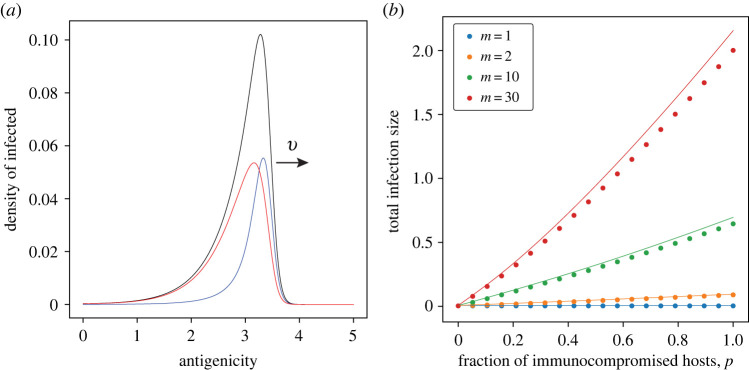
Composition of immunocompromised and immune-competent hosts in the infected population in a stable travelling wave in antigenic space. (*a*) The travelling wave profiles of infected hosts over antigenic space in a stable travelling wave of antigenic escape. The black curve shows the total infected density, and the red and the blue curve represents the infected density of immunocompromised and immune-competent hosts, respectively. (*b*) The total infection size Φ at a stable travelling wave is plotted against the fraction *p* of immunocompromised hosts. The dots are the results of numerical simulation of (2.1) and solid curves are the prediction from (3.3). Different colours are for different *m* = *γ*_0_/*γ*_1_ values. *m* = *γ*_0_/*γ*_1_ = 10 and *p* = 0.15 in (*a*). The other parameters are *β* = 1.1, *γ*_0_ = 1, *D* = 0.001. (Online version in colour.)

Each stable travelling wave of antigenic variants in host populations of heterogeneous immune competency (figure 1*b*) is decomposed into that in immune-competent hosts (blue) and immunocompromised hosts (red) (figure 3*a*). The asymptotic value of the total number of infected hosts at time *t*, $\Phi =\int_{-\infty }^{\infty }(I_{0}(t,x)+I_{1}(t,x))\;dx$, that quantifies the impact to the infectious disease caused by a continuously immune-evading pathogen, is determined from the following relationship, similar to (2.3) (see Methods for the deviation):

3.3 $$\begin{array}{c} \Phi =\frac{v}{{\left\langle \gamma \right\rangle }_{H}}\left(1-\exp\left[-\frac{\beta }{v}\Phi \right]\right), \end{array}$$

where *v* is the wave speed for a homogeneous host population defined in (3.1). Comparing this with (2.3) for a homogeneous host population, we see that the wave speed *v*_0_ in a homogeneous population is replaced by *v* in a heterogeneous population, and the recovery rate *γ*_0_ of immune-competent hosts is replaced by the harmonic mean of recovery rates, 〈*γ*〉*_H_* = ((1 − *p*)/*γ*_0_ + *p*/*γ*_1_)^−1^ in the heterogeneous host population (figure 3*b*). In addition, the ratio of infected immunocompromised hosts, $\Phi_{1}=\int_{-\infty }^{\infty }I_{1}(t,x)\;dx$ to that of immunocompetent hosts, $\Phi_{0}=\int_{-\infty }^{\infty }I_{0}(t,x)\;dx$, in a stationary travelling wave is shown as follows:

3.4 $$\begin{array}{c} \frac{\Phi_{1}}{\Phi_{0}}=\frac{\;p/\gamma_{1}}{(1-p)/\gamma_{0}}=m\frac{\;p}{1-p}. \end{array}$$

This implies that immunocompromised hosts are at *m* = *γ*_0_/*γ*_1_ times higher risk of infection than immune-competent hosts. Thus, not only do immunocompromised hosts disproportionately accelerate the speed of antigenic evolution of pathogens in the entire population, but they each bear a higher risk of infection than hosts with normal immune competency, suggesting the crucial importance of caring for immunocompromised hosts to protect the entire population and themselves.

### (d) Control measures for suppressing antigenic escape

**Figure 4.**
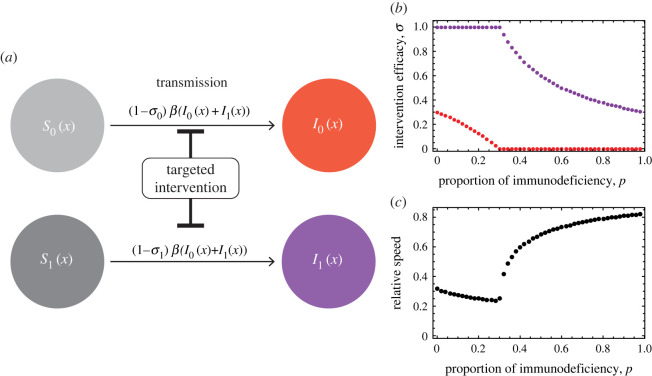
The optimal intervention scheme in the host population with heterogeneous immune competency. (*a*) A graphical representation of the model of targeted intervention strategy. (*b*) Under an economic cost-induced trade-off between *σ*_0_ and *σ*_1_, (1 − *p*)*σ*_0_ + *pσ*_1_ = *c*, the optimal intervention $\left(\sigma_{0}^{*},\sigma_{1}^{*}\right)$ that minimizes the speed of antigenic escape evolution is shown for varied proportions of immunocompromised hosts. The purple and red dots show the optimal degrees, *σ*_0_ and *σ*_1_, of reducing susceptibilities of normal and immunocompromised hosts, respectively. (*c*) The relative speed *v*^′^/*v* under optimal intervention to that without intervention. The parameters are *β* = 1.5, *γ*_0_ = 1, *m* = 10, *D* = 0.001 and *c* = 0.3 (the economic budget for control is just enough to reduce the susceptibility of all individuals by 30%, or to perfectly reduce the susceptibility of 30% of the population, or a mixture of the efficiency and coverage). (Online version in colour.)

As a strategy to slow antigenic escape, targeted interventions that concentrate countermeasures (e.g. vaccines, quarantine, etc.) on specific populations are considered (figure 4*a*). Here, intervention is assumed to reduce susceptibility. Let *σ_i_* be the efficiency of the intervention on host class *i*, assuming that the susceptibility of the particular host population *i* is reduced to be 1 − *σ_i_* times (*σ_i_* ≤ 1) that before the intervention. The corresponding model for this case can be written as follows:

3.5 $$\begin{array}{rl} \frac{\partial S_{0}(t,x)}{\partial t}= & -\beta (1-\sigma_{0})S_{0}(t,x)(I_{0}(t,x)+I_{1}(t,x)),\\ \frac{\partial S_{1}(t,x)}{\partial t}= & -\beta (1-\sigma_{1})S_{1}(t,x)(I_{0}(t,x)+I_{1}(t,x)),\\ \frac{\partial I_{0}(t,x)}{\partial t}= & \beta (1-\sigma_{0})S_{0}(t,x)(I_{0}(t,x)+I_{1}(t,x))-\gamma_{0}I_{0}(t,x)+D\frac{\partial^{2}I_{0}(t,x)}{\partial x^{2}}\\ \mathrm{and}\;\frac{\partial I_{1}(t,x)}{\partial t}= & \beta (1-\sigma_{1})S_{1}(t,x)(I_{0}(t,x)+I_{1}(t,x))-\gamma_{1}I_{1}(t,x)+D\frac{\partial^{2}I_{1}(t,x)}{\partial x^{2}}. \end{array}\}$$

This model contains heterogeneity in both recovery rate and susceptibility. We also calculate the rate of antigenic change in this model analytically as before (Methods), allowing us to examine how changes in susceptibility due to targeted intervention affect the rate of antigenic escape evolution.

A targeted intervention scenario is considered in which the intervention is applied to both immunocompromised and immune-competent hosts, but the degree of intervention varies between classes (figure 4*b*). The presence of immunocompromised hosts certainly accelerates the rate of antigenic escape evolution; however, because the proportion of immunocompromised hosts is usually smaller than that of immune-competent hosts, whether intervention should be focused on the small number of immunocompromised hosts or the large number of immune-competent hosts is unclear. We assume that the intervention has an economic cost *c*, which is given by the product of the degree of the suppression effect *σ_i_* and the size of the target, 1 − *p* for *i* = 0 and *p* for *i* = 1:

3.6 $$\begin{array}{c} c=\sigma_{0}(1-p)+\sigma_{1}p. \end{array}$$

If we denote the speed of antigenic evolution under intervention as *v*^′^, the most efficient intervention for a fixed economical cost (3.6) is to minimize its ratio, *v*^′^/*v*, to the speed *v* before intervention. Thus, we calculated the optimal targeted intervention strategy, (*σ*_0_, *σ*_1_), that minimizes *v*^′^/*v* under the constraint (3.6) for varied proportions *p* of immunocompromised hosts (figure 4*b*). When *p* is less than the threshold *p_c_* = *c* (the threshold is *p_c_* = 0.3; figure 4*b*), the optimal intervention strategy $\left(\sigma_{0}^{*},\sigma_{1}^{*}\right)$ is to try to maximally reduce the susceptibility of immunocompromised hosts, while reducing the susceptibility of immune-competent hosts as far as the cost allows: $\sigma_{0}^{*}=(c-p)/(1-p)>0$ and $\sigma_{1}^{*}=1$ (figure 4*b*). By contrast, when *p* is greater than the threshold, the optimal intervention focuses only on reducing the susceptibility of immunocompromised hosts as far as the cost allows and completely ignoring the intervention for immune-competent hosts: $\sigma_{0}^{*}=0$ and $\sigma_{1}^{*}=c/p>0$ (figure 4*b*). These results show that the optimal intervention strategy prioritizes the intervention focusing on immunocompromised hosts, indicating that even if immunocompromised hosts exist only in small numbers, preferential interventions aimed at them can efficiently reduce the rate of antigenic evolution. Interestingly, the relative wave speed *v*^′^/*v* under optimal intervention control shows qualitatively different behaviours below and above *p* = *p_c_* (figure 4*c*). Namely, if *p* is less than the threshold, the relative wave speed *v*^′^/*v* decreases with increasing *p*, indicating that the efficiency of the intervention increases with the number of immunocompromised hosts (figure 4*c*). By contrast, if *p* is above the threshold value, the speed of antigenic escape rapidly increases and approaches the value without intervention as *p* increases, indicating that the intervention rapidly loses its effectiveness as the number of immunocompromised hosts increases.

To be more practical, we need to carefully consider the more detailed relationship between targeted intervention measures, their effectiveness in reducing susceptibility and their economic costs. However, this example illustrates the applicability of our model to design effective intervention policies to slow pathogenic immune escape.

## 4. Discussion

The ongoing epidemic of SARS-CoV-2 has been accompanied by the emergence of several variants, and attention has focused on the evolutionary causes of such variants [2,3]. One mechanism that has been suggested is the presence of immunocompromised hosts in the population [13,40,41]. In this study, we theoretically examine the impact of host heterogeneity in immune competency on the speed of antigenic escape of pathogens at the population level. Given that variation in immune competency is characterized by differences in recovery rates, we developed a model to represent the dynamics of antigenic drift in a population with heterogeneous recovery rates and derived the speed of the accumulation of antigenic escape mutants of a pathogen circulating in the population. We then examined the effect of the presence of immunocompromised hosts on the accumulation of the antigenic escape mutants of the pathogen. The model reveals that the presence of immunocompromised hosts results in a faster accumulation of antigen-escaping mutations in the pathogen. In particular, we reveal that even if only a small proportion of immunocompromised hosts is present, the impact on the rate of antigenic evolution is significant. This emphasizes the importance of the presence of immunocompromised hosts not only on antigenic evolutionary dynamics at the individual level, but also at the population level. These results highlight the importance of careful surveillance and countermeasures for immunocompromised hosts [42].

The presence of immunocompromised hosts not only sensitively accelerates the speed of antigenic escape of the pathogen, but also increases the magnitude of pathogen prevalence in the entire population. Furthermore, immunocompromised hosts are to be found in the infected population at a much higher rate than in their original composition.

### (a) Why do hosts with weak immune competency accelerate antigenic escape?

**Figure 5.**
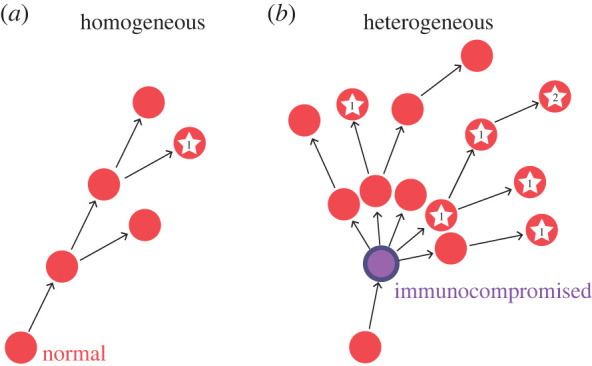
A graphical explanation of the acceleration of antigenic escape by immunocompromised hosts. Transmission chains in the same time interval in homogeneous (*a*) and heterogeneous host populations (*b*) are shown. The red and purple circle represents infected normal and immunocompromised hosts, respectively. The white star represents the host infected by antigenic escaping mutants, and the number within the star gives the accumulated number of escaping mutations. (Online version in colour.)

Why do immunocompromised hosts accelerate the speed of antigenicity evolution? The reason is simply attributed to the fact that immunocompromised hosts increase the pathogen growth rate in the population. We illustrate this by comparing transmission chains in a population consisting only of normal hosts (figure 5*a*) and a population where some of hosts are immunocompromised (figure 5*b*). An immunocompromised host (purple circle in figure 5*b*) causes on average more infections than a normal host (red circle) per unit time due to his/her smaller recovery rate, resulting in faster growth of the infected population in the host population with immunocompromised hosts (figure 5*b*) than the population without (figure 5*a*). The faster growth of the infected population also increases the likelihood that viruses will accumulate antigenic escaping mutations (stars in figure 5). In fact, initial growth rate of the pathogen in the homogeneous host population consisting of only normal immunocompetent hosts is given by *r*_0_ = *β* − *γ*_0_. The speed of antigenic evolution in a homogeneous host population, *v*_0_, is then given by the equation $v_{0}=2\sqrt{r_{0}D}$ as shown in (2.2). By contrast, in the heterogeneous host population, the initial growth rate of the infected population, *r*, is calculated from the largest eigenvalue of the linearized epidemiological dynamics, and the speed of antigenic evolution is also given by $v=2\sqrt{rD}$ (see Method). We can show that, in the presence of immunocompromised hosts, *r* is always larger than *r*_0_. Thus, our theory suggests that immunocompromised hosts contribute to the increased rate of antigenic evolution by speeding up the growth rate of mutant strains and enhancing rapid epidemic turnover.

The above explanation is based purely on our theoretical framework and from the epidemiological perspective. Other potential mechanisms may also contribute to the promotion of viral antigenic evolution within infected immunocompromised hosts. For example, the lack of selection pressure by the immune system may allow the survival of viral variants that are not viable under normal host immune pressure, which potentially increases the chance for antigenicity-changing mutations to accumulate. Another possibility is that adaptation to multiple tissues that are more likely to occur under long-term infection in immunocompromised hosts may promote viral diversity including those contributing to antigenicity escapes. For example, SARS-CoV-2 is known to have different mutations in the upper and lower respiratory tracts in immunocompromised hosts [18] and this suggests diversification through adaptation to different niches. Still another possibility is that the increased viral load in immunocompromised hosts may promote viral recombination by causing multiple infections, though to our knowledge, there have been no reports of recombination in immunocompromised patients. These possible factors influencing the evolution of pathogen antigenicity in the presence of heterogeneity in host immune competency need to be tested in the future.

### (b) Factors leading to heterogeneous recovery rates

The strength of our study is that it provides an analytical and quantitative prediction of the extent to which the heterogeneity in host immune competency affects antigenic escape. Specifically, we have revealed that the presence of hosts with longer infectious periods (lower recovery rates) could have a significant impact on the speed of antigenic evolution. To apply our theoretical results, data on the distribution of recovery rates in the host population is therefore particularly important. Factors that may cause differences in recovery rates are not limited to immunodeficiency. Other factors including age structure and vaccine dose conditions also affect host recovery rates. For example, older individuals recover more slowly than younger individuals [43], and unvaccinated individuals may experience slower recovery than vaccinated individuals [44–46]. Thus, understanding the distribution of recovery rates shaped by these diverse host factors is crucial to antigenic evolution of pathogens.

### (c) Effective intervention strategy to suppress antigenic evolution

We also applied our model to seek effective intervention strategies against pathogens capable of escaping host immunity. The targeted intervention to particular subpopulations is promising as any intervention including vaccination and behavioural regulation is accompanied by economic cost and overwhelming medical resources [47–51]. Suppressing antigenic changes to slow the emergence of new immune-escaping variants is crucial because it allows time to develop vaccines against new variants and other countermeasures, such as antiviral drugs. In addition, slowing the rate of antigenic drift could lead to the containment of the epidemic with the existing vaccines. In considering effective measures, host heterogeneity in immune competency again becomes important. Our theory shows that effective measures in targeted interventions should focus on caring for hosts with weak immune competency (figure 4*b*). Because these hosts are at higher risk of infection than other hosts, a prioritized countermeasure protecting immunocompromised hosts has the advantage of protecting the entire population by slowing antigen escape. Because immunocompromised hosts are generally uncommon and specific interventions for such hosts are highly effective in slowing antigenic drift, priority measures targeted at them can be effective at relatively low economic and medical costs. While these results are based on several assumptions as to the effectiveness of targeted intervention and its cost, they indicate the potential to provide a basis for applying our model or an extended version of it to develop effective intervention strategies as shown below.

### (d) Possible interventions targeting immunocompromised hosts

Antibodies induced by vaccination normally protect vaccinees by rapidly eliminating viruses when infected. Unfortunately, immunocompromised patients have low seroconversion rates and low antibody titres after vaccination [52]. However, even in immunocompromised hosts, multiple vaccination doses have been suggested to increase antibody titres and seroconversion rates [52]. Prioritizing immunocompromised patients for multiple doses of vaccine could be an effective strategy to slow down the antigenic escape of viruses because, as our theory suggests, immunocompromised hosts have a disproportionally high contribution to the speed. Even more promising would be antiviral drug treatments against immunocompromised hosts [42], as its mechanism of inhibiting viral replication mechanism does not rely on the immune competency of patients.

### (e) Comparison to previous studies

Viral adaptation in host populations with heterogeneous immune competency is beginning to attract attention. For example, Smith & Ashby [37] numerically investigated the effect of immunocompromised hosts on antigenic evolution. Their focus is on the joint process of crossing the fitness valley and antigenicity escape. Under the assumption that there is some correlation between pathogen infectivity and antigenicity, they numerically showed that the presence of immunocompromised hosts allows pathogens to overcome an otherwise insurmountable fitness valley. Although our model is similar to theirs except for their additional assumption on a transmissibility–antigenicity trade-off, our results give more general and analytical predictions for the rate and shape of antigenic evolution.

In the context of vaccine administration, viral adaptation in heterogeneous host populations is also of growing interest. Recently, several studies have evaluated optimal vaccination policies to reduce the emergence of mutations that escape vaccines, taking into account host heterogeneity in the vaccination dose [47,48,53]. For example, Gandon & Lion [47] showed under various vaccination scenarios, there could be discrepancy in optimal vaccination strategies between the epidemiological perspective (i.e. limiting the size of the epidemic) and the evolutionary perspective (i.e. limiting viral evolution). The resolution of such a discrepancy by the extension of the present analytical theory would be an interesting future study.

### (f) Other heterogeneities than recovery rate

Our theory can consider not only heterogeneity in recovery rates, but also more general heterogeneity, such as susceptibility and infectivity, and the rate of antigenic escape can be determined analytically (see Methods). Hosts have a variety of characteristics, including age, sex and immune status. Heterogeneity in multiple host parameters, such as recovery rates and susceptibility, may be related to such host conditions. For example, heterogeneity in vaccination status creates heterogeneity in both susceptibility and recovery rates [44,46]. As noted previously, several studies addressed host heterogeneity in the vaccination doses to determine vaccination strategies to prevent viral adaptation to vaccines [47,48,53]. Our theory may also be useful in considering interventions to control vaccine escape mutants by considering vaccine-induced host heterogeneity. An interesting extension of our model for future research would be to determine how various intervention strategies affect the speed of antigen drift while accounting for host heterogeneity.

### (g) Theoretical implications

Finally, we briefly situate this study within a theoretical context. In evolutionary ecology, much attention has been paid to the effects of heterogeneity on evolution in many contexts [54]. This study theoretically addresses the speed of travelling wave solutions in heterogeneous populations. Although theoretical research on travelling waves is found in many evolutionary and ecological studies since Fisher's work on the geographical spread of an organism [34], the studies of travelling waves in heterogeneous populations remain scarce (but see Shigesada & Kawasaki, [55]). Thus, our theoretical approach holds promise for further development concerning directional evolution in heterogeneous populations.

## 5. Methods

### (a) Analytical calculation of wave speed in a host population with heterogeneous recovery

A travelling wave solution to the partial differential equations of our model (1) can be rewritten as a system of ordinary differential equations of *S_i_*(*z*) = *S_i_*(*t*, *x*) and *I_i_*(*z*) = *I_i_*(*t*, *x*) by introducing a moving coordinate with the same speed *v* as the travelling wave: *z* = *x* – *vt*:

5.1*a* $$vS_{0}^{\prime }(z)-\beta S_{0}(z)(I_{0}(z)+I_{1}(z))=0,$$

5.1*b* $$vS_{1}^{\prime }(z)-\beta S_{1}(z)(I_{0}(z)+I_{1}(z))=0,$$

5.1*c* $$DI_{0}^{\prime \prime }(z)+vI_{0}^{\prime }(z)+\beta S_{0}(z)(I_{0}(z)+I_{1}(z))-\gamma_{0}I_{0}(z)=0$$

5.1*d* $$\mathrm{and}\;DI_{1}^{\prime \prime }(z)+vI_{1}^{\prime }(z)+\beta S_{1}(z)(I_{0}(z)+I_{1}(z))-\gamma_{1}I_{1}(z)=0,$$

where ′ = *d*/*dz* and ′′ = *d*^2^/*dz*^2^ denotes the differentiations with respect to *z*. We linearize at the front end of the travelling wave of antigenic variants where all hosts are susceptible, *S*_0_(*z*) = 1 − *p* and *S*_1_(*z*) = *p*:

5.2*a* $$DI_{0}^{\prime \prime }(z)+vI_{0}^{\prime }(z)+\beta (1-p)(I_{0}(z)+I_{1}(z))=0$$

and

5.2*b* $$DI_{1}^{\prime \prime }(z)+vI_{1}^{\prime }(z)+\beta p(I_{0}(z)+I_{1}(z))=0.$$

We then substitute exponential forms *I*_0_(*z*) = *ξ*_0_*e*^−*λ*^*^z^* and *I*_1_(*z*) = *ξ*_1_*e*^−*λ*^*^z^* where *λ* > 0 is the rate of exponential decay of travelling waves towards their frontal end and *ξ_i_* > 0 is the positive constant representing the relative weights of immune-competent and immunocompromised hosts in the frontal end of the travelling wave. Substituting these into (5.2) yields:

5.3 $$\begin{array}{c} \left(\begin{array}{cc} D\lambda^{2}-v\lambda +a_{0} & b_{1}\\ b_{0} & D\lambda^{2}-v\lambda +a_{1} \end{array}\right)\left(\begin{array}{c} \xi_{0}\\ \xi_{1} \end{array}\right)=\left(\begin{array}{c} 0\\ 0 \end{array}\right), \end{array}$$

where *a_i_* denotes the initial growth rate within class *i*, and *b_i_* denotes the contribution of class *i* to the other class

5.4 $$\begin{array}{c} a_{0}=\beta (1-p)-\gamma_{0},\;a_{1}=\beta p-\gamma_{1},\;b_{0}=\beta p,\;b_{1}=\beta (1-p). \end{array}$$

To obtain a non-trivial solution, ${\left(\xi_{0},\xi_{1}\right)}^{⊤}\neq {\left(0,0\right)}^{⊤}$, to (5.3), the matrix in the left-hand side of (5.3) must be non-singular, and hence, its determinant must vanish:

5.5 $$\begin{array}{c} (D\lambda^{2}-v\lambda +a_{0})(D\lambda^{2}-v\lambda +a_{1})-b_{0}b_{1}=0. \end{array}$$

This is a quadratic equation for *χ* = *vλ* − *Dλ*^2^, *χ*^2^ − (*a*_0_ + *a*_1_)*χ* + *a*_0_*a*_1_ − *b*_0_*b*_1_ = 0, and is solved as follows:

5.6 $$\begin{array}{c} \chi_{\pm }=\frac{1}{2}\left[a_{0}+a_{1}\pm \sqrt{{\left(a_{0}-a_{1}\right)}^{2}+4b_{0}b_{1}}\right]. \end{array}$$

For the solution to be biologically feasible, both *ξ*_0_ and *ξ*_1_ must be positive, which means that only *χ*_+_ corresponds to a biologically feasible solution. This relationship, *χ* = *vλ* − *Dλ*^2^ = *χ*_+_, can be rewritten as

5.7 $$\begin{array}{c} v=\frac{\chi_{+}}{\lambda }+D\lambda . \end{array}$$

This is the dispersion relationship between the wave speed *v* and the exponential decay rate *λ* of the travelling wave tail. In scalar equations, the travelling wave solution exists only for the wave speed *v* ≥ *v*_min_, where *v*_min_ is the minimum wave speed as a function of *λ*, and the stable wave solution for adequate initial distributions (e.g. the initial distribution is confined to the left of a certain position) has *v*_min_ as its speed. In a vector-valued system as in our model (which has a pair of distributions (*I*_0_(*z*), *I*_1_(*z*))), the same principle is mathematically proven under certain conditions [34,38,55,56]; however, in some cases linearization of the system does not give the speed of the stable travelling wave [36,57]. We have confirmed that the minimum wave speed in the linearized system (5.2) fits the speed observed in the numerical simulation of our model (1).

The wave speed *v*_min_ that minimizes (5.7) as a function of *λ* is

5.8 $$\begin{array}{c} v_{\min}=2\sqrt{\chi_{+}D}=\sqrt{2D\left[a_{0}+a_{1}\pm \sqrt{{\left(a_{0}-a_{1}\right)}^{2}+4b_{0}b_{1}}\right]}. \end{array}$$

By substituting (5.4) into (5.8), we obtain analytical expression (3.1) in the main text.

The formula (5.8) or $v=2\sqrt{\chi_{+}D}$ for the speed of antigenic escape in a heterogeneous host population has a similar form to the speed $v=2\sqrt{r_{0}D}$ in a homogeneous host population without any immunocompromised hosts, where *r*_0_ = *β* − *γ*_0_ is the initial growth rate of the infected population in the homogeneous case. This similarity is not just formal. We will see below that *χ*_+_ gives the initial pathogen growth rate in the heterogeneous host population. At the beginning of the epidemic with an initial antigenicity variant, epidemiological dynamics is given by the linearized system:

5.9 $$\begin{array}{rl} \left(\begin{array}{c} \frac{dI_{0}}{dt}\\ \frac{dI_{1}}{dt} \end{array}\right) & =\left(\begin{array}{cc} \beta (1-p)-\gamma_{0} & \beta (1-p)\\ \beta p & \beta p-\gamma_{1} \end{array}\right)\left(\begin{array}{c} I_{0}\\ I_{1} \end{array}\right)\\ & =\left(\begin{array}{cc} a_{0} & b_{1}\\ b_{0} & a_{1} \end{array}\right)\left(\begin{array}{c} I_{0}\\ I_{t} \end{array}\right)=M\left(\begin{array}{c} I_{0}\\ I_{t} \end{array}\right). \end{array}$$

The largest eigenvalue *r* of matrix *M*, which gives the initial growth rate of pathogen, is shown to be identical to *χ*_+_:

$$r=\frac{1}{2}\left[a_{0}+a_{1}+\sqrt{{\left(a_{0}-a_{1}\right)}^{2}+4b_{0}b_{1}}\right]=\chi_{+}.$$

### (b) Analytical calculation of total epidemic size and the ratio of infected immunocompromised hosts

In this section, we derive (3.3) and (3.4) in the main text. Integrating both sides of (5.1*a*) from *z* to ∞ give

$$\begin{array}{rl} \int_{z}^{\infty }\frac{1}{S_{0}(z^{\prime })}\;\;\frac{dS_{0}(z^{\prime })}{dz^{\prime }}\;dz^{\prime } & ={\left[\log S_{0}(z^{\prime })\right]}_{z}^{\infty }=\log\frac{S_{0}(\infty )}{S_{0}(z)}=\log\frac{1-p}{S_{0}(z)}\\ & =\frac{\beta }{v}\int_{z}^{\infty }(I_{0}(z^{\prime })+I_{1}(z^{\prime }))\;dz^{\prime }, \end{array}$$

Where we used *S*_0_(∞) = 1 − *p*. This yields the following:

5.10*a* $$\begin{array}{c} S_{0}(z)=(1-p)\exp\left[-\frac{\beta }{v}\int_{z}^{\infty }(I_{0}(z^{\prime })+I_{1}(z^{\prime }))\;dz^{\prime }\right]. \end{array}$$

In the same vein, we obtain the following from (5.1*b*):

5.10*b* $$\begin{array}{c} S_{1}(z)=p\exp\left[-\frac{\beta }{v}\int_{z}^{\infty }(I_{0}(z^{\prime })+I_{1}(z^{\prime }))\;dz^{\prime }\right]. \end{array}$$

Substituting (5.9) into (5.1*c*) and (5.1*d*) to eliminate *S*_0_(*z*) and *S*_1_(*z*), and noting the following:

$$\begin{array}{rl} \beta S_{0}(z)(I_{0}(z)+I_{1}(z)) & =\beta (1-p)\exp\left[-\frac{\beta }{v}\int_{z}^{\infty }\left(I_{0}(z^{\prime })+I_{1}(z^{\prime })\right)\;dz^{\prime }\right]\\ \left(I_{0}(z)+I_{1}(z)\right) & =(1-p)v\frac{d}{dz}\exp\left[-\frac{\beta }{v}\int_{z}^{\infty }(I_{0}(z^{\prime })+I_{1}(z^{\prime }))\;dz^{\prime }\right],\\ \beta S_{1}(z)(I_{0}(z)+I_{1}(z)) & =pv\frac{d}{dz}\exp\left[-\frac{\beta }{v}\int_{z}^{\infty }(I_{0}(z^{\prime })+I_{1}(z^{\prime }))\;dz^{\prime }\right], \end{array}$$

we obtain:

$$\begin{array}{r} \frac{d}{dz}\left[D\frac{dI_{0}(z)}{dz}\;+vI_{0}(z)+(1-p)v\exp\left[-\frac{\beta }{v}\int_{z}^{\infty }\left(I_{0}(z^{\prime })+I_{1}(z^{\prime })\right)\;dz^{\prime }\right]-\gamma_{0}I_{0}(z)\right]=0\\ \frac{d}{dz}\left[D\frac{dI_{1}(z)}{dz}\;+vI_{1}(z)+pv\exp\left[-\frac{\beta }{v}\int_{z}^{\infty }\left(I_{0}(z^{\prime })+I_{1}(z^{\prime })\right)\;dz^{\prime }\right]-\gamma_{1}I_{1}(z)\right]=0. \end{array}$$

Integrating both sides from − ∞ to ∞ and noting *I_i_*( ± ∞) = 0 and $I_{i}^{\prime }(\pm \infty )=0$, we then have:

$$\begin{array}{rl} & (1-p)v\left(1-\exp\left[-\frac{\beta }{v}\int_{-\infty }^{\infty }(I_{0}(z^{\prime })+I_{1}(z^{\prime }))\;dz^{\prime }\right]\right)-\gamma_{0}\int_{-\infty }^{\infty }I_{0}(z)\;dz=0\\ & pv\left(1-\exp\left[-\frac{\beta }{v}\int_{-\infty }^{\infty }\left(I_{0}(z^{\prime })+I_{1}(z^{\prime })\right)\;dz^{\prime }\right]\right)-\gamma_{1}\int_{-\infty }^{\infty }I_{1}(z)\;dz=0. \end{array}$$

Thus, the following equations can be used for $\Phi_{0}=\int_{-\infty }^{\infty }I_{0}(z)\;dz$ and $\Phi_{1}=\int_{-\infty }^{\infty }I_{1}(z)\;dz$:

5.11*a* $$\Phi_{0}=\frac{(1-p)v}{\gamma_{0}}\left(1-\exp\left[-\frac{\beta }{v}(\Phi_{0}+\Phi_{1})\right]\right),$$

and

5.11*b* $$\Phi_{1}=\frac{\;pv}{\gamma_{1}}\left(1-\exp\left[-\frac{\beta }{v}(\Phi_{0}+\Phi_{1})\right]\right).$$

By taking the ratio of both sides of (5.10*b*) to those of (5.10*a*), we obtain (7) in the text:

$$\frac{\Phi_{1}}{\Phi_{0}}=\frac{\;p/\gamma_{1}}{(1-p)/\gamma_{0}}=m\frac{\;p}{1-p}.$$

By adding (5.10*a*) and (5.10*b*), we obtain the equation (3.3) for the total epidemic size, Φ = Φ_0_ + Φ_1_ in the main text:

$$\begin{array}{c} \Phi =v\left(\frac{1-p}{\gamma_{0}}+\frac{\;p}{\gamma_{1}}\right)\left(1-\exp\left[-\frac{\beta }{v}\Phi \right]\;\right)\\ =\frac{v}{\gamma_{H}}\left(1-\exp\left[-\frac{\beta }{v}\Phi \right]\right), \end{array}$$

where 〈*γ*〉*_H_* = ((1 − *p*)/*γ*_0_ + *p*/*γ*_1_)^−1^ represents the harmonic mean of recovery rates.

### (c) Analytical calculation of wave speed in a general heterogeneous host population

If heterogeneity is present in susceptibility and transmissibility as well as in recovery rates, model (1) is extended to the following:

5.12 $$\begin{array}{rl} \frac{\partial S_{0}(t,x)}{\partial t}= & -\beta s_{0}S_{0}(t,x)(t_{0}I_{0}(t,x)+t_{1}I_{1}(t,x)),\\ \frac{\partial S_{1}(t,x)}{\partial t}= & -\beta s_{1}S_{1}(t,x)(t_{0}I_{0}(t,x)+t_{1}I_{1}(t,x)),\\ \frac{\partial I_{0}(t,x)}{\partial t}= & \beta s_{0}S_{0}(t,x)(t_{0}I_{0}(t,x)+t_{1}I_{1}(t,x))-\gamma_{0}I_{0}(t,x)+D\frac{\partial^{2}I_{0}(t,x)}{\partial x^{2}}\\ \mathrm{and}\;\frac{\partial I_{1}(t,x)}{\partial t}= & \beta s_{1}S_{1}(t,x)(t_{0}I_{0}(t,x)+t_{1}I_{1}(t,x))-\gamma_{1}I_{1}(t,x)+D\frac{\partial^{2}I_{1}(t,x)}{\partial x^{2}}, \end{array}$$

where *s_i_*, *t_i_* and *γ_i_* represent the susceptibility, transmissibility and recovery rate of class *i* hosts, respectively.

We can obtain the analytical formulae in this model by using the above procedure. Briefly, linearizing this system at the frontal end of the wave, we essentially get the same equation as (5.12):

5.13 $$\begin{array}{c} \left(\begin{array}{cc} D\lambda^{2}-v\lambda +A_{0} & B_{1}\\ B_{0} & D\lambda^{2}-v\lambda +A_{1} \end{array}\right)\left(\begin{array}{c} \xi_{0}\\ \xi_{1} \end{array}\right)=\left(\begin{array}{c} 0\\ 0 \end{array}\right), \end{array}$$

where *A*_0_ = *βs*_0_*t*_0_(1 − *p*) − *γ*_0_, *A*_1_ = *βs*_1_*t*_1_*p* − *γ*_1_, *B*_0_ = *βs*_1_*t*_0_*p* and *B*_1_ = *βs*_0_*t*_1_(1 − *p*). We then obtain the formula for the wave speed:

5.14 $$\begin{array}{c} v=\sqrt{2D\left[A_{0}+A_{1}\pm \sqrt{{\left(A_{0}-A_{1}\right)}^{2}+4B_{0}B_{1}}\right]}. \end{array}$$

In the main text, we consider intervention strategies that target subpopulations in a specific manner. Intervention strategy (*σ*_0_, *σ*_1_) changes the susceptibility of class *i* hosts to 1 − *σ_i_* times the susceptibility before intervention. In this situation, *A*_0_ = *β*(1 − *σ*_0_)(1 − *p*) − *γ*_0_, *A*_1_ = *β*(1 − *σ*_1_)*p* − *γ*_1_, *B*_0_ = *β*(1 − *σ*_1_)*p*, *and B*_1_ = *β*(1 − *σ*_0_)(1 − *p*). Applying these to (5.13) gives the speed of antigenic evolution under the intervention strategies.

## Data Availability

Supplementary information is provided in the electronic supplementary material [58].
